# European Multicentre Tics in Children Studies (EMTICS): protocol for two cohort studies to assess risk factors for tic onset and exacerbation in children and adolescents

**DOI:** 10.1007/s00787-018-1190-4

**Published:** 2018-07-07

**Authors:** Anette Schrag, Davide Martino, Alan Apter, Juliane Ball, Erika Bartolini, Noa Benaroya-Milshtein, Maura Buttiglione, Francesco Cardona, Roberta Creti, Androulla Efstratiou, Maria Gariup, Marianthi Georgitsi, Tammy Hedderly, Isobel Heyman, Immaculada Margarit, Pablo Mir, Natalie Moll, Astrid Morer, Norbert Müller, Kirsten Müller-Vahl, Alexander Münchau, Graziella Orefici, Kerstin J. Plessen, Cesare Porcelli, Peristera Paschou, Renata Rizzo, Veit Roessner, Markus J. Schwarz, Tamar Steinberg, Friederike Tagwerker Gloor, Zsanett Tarnok, Susanne Walitza, Andrea Dietrich, Pieter J. Hoekstra, EMTICS Collaborative Group, Zacharias Anastasiou, Alan Apter, Valentina Baglioni, Juliane Ball, Erika Bartolini, Noa Benaroya-Milshtein, Benjamin Bodmer, Emese Bognar, Bianka Burger, Judith Buse, Maura Buttiglione, Francesco Cardona, Marta Correa Vela, Roberta Creti, Andrea Dietrich, Nanette M. Debes, Androulla Efstratiou, Maria Cristina Ferro, Carolin Fremer, Blanca Garcia-Delgar, Maria Gariup, Marianthi Georgitsi, Mariangela Gulisano, Annelieke Hagen, Julie Hagstrøm, Tammy J. Hedderly, Isobel Heyman, Pieter J. Hoekstra, Chaim Huyser, Monica Imperi, Iordanis Karagiannidis, Giovanni Laviola, Simone Macri, Marcos Madruga-Garrido, Immaculada Margarit, Anna Marotta, Davide Martino, Ute C. Meier, Pablo Mir, Natalie Moll, Astrid Morer, Norbert Müller, Kirsten Müller-Vahl, Alexander Münchau, Peter Nagy, Valeria Neri, Thaïra J. C. Openneer, Graziella Orefici, Peristera Paschou, Alessandra Pellico, Onofrio Petruzzelli, Kerstin J. Plessen, Cesare Porcelli, Marina Redondo, Renata Rizzo, Paolo Roazzi, Veit Roessner, Daphna Ruhrman, Jaana M. L. Schnell, Anette Schrag, Gregor A. Schütze, Markus J. Schwarz, Paola Rosaria Silvestri, Liselotte Skov, Tamar Steinberg, Sara Stöber, Friederike Tagwerker Gloor, Marco Tallon, Zsanett Tarnok, Victoria L. Turner, Susanne Walitza, Elif Weidinger, Martin L. Woods

**Affiliations:** 1 Department of Clinical Neurosciences, UCL Institute of Neurology, University College London, London, UK; 2 Department of Clinical Neurosciences, University of Calgary, Calgary, Canada; 3 Child and Adolescent Psychiatry Department, Schneider Children’s Medical Center of Israel, Affiliated to Sackler Faculty of Medicine, Tel Aviv University, Petah-Tikva, Israel; 4 Clinic of Child and Adolescent Psychiatry and Psychotherapy, University of Zurich, Zurich, Switzerland; 5 GSK, Siena, Italy; 6 Department of Biological Sciences and Human Oncology, Medical School, University of Bari “Aldo Moro”, Bari, Italy; 7 Department of Human Neurosciences, University La Sapienza of Rome, Rome, Italy; 8 Department of Infectious Diseases, Istituto Superiore di Sanità, Rome, Italy; 9 WHO Global Collaborating Centre for Reference and Research on Diphtheria and Streptococcal Infections, Reference Microbiology, Directorate National Infection Service, Public Health England, London, UK; 10 University of Barcelona, Barcelona, Spain; 11 Intensive Inpatient Unit, Copenhagen Psychiatric Center, Copenhagen, Denmark; 12 Department of Molecular Biology and Genetics, Democritus University of Thrace, Alexandroupoli, Greece; 13 Department of Medicine, Aristotle University of Thessaloniki, Thessaloníki, Greece; 14 Evelina London Children’s Hospital GSTT, Kings Health Partners AHSC, London, UK; 15 Great Ormond Street Hospital for Children, UCL Institute of Child Health, London, UK; 16 Unidad de Trastornos del Movimiento, Servicio de Neurología y Neurofisiología Clinica, Instituto de Biomedicina de Sevilla (IBiS), Hospital Universitario Virgen del Rocio/CSIC/Universidad de Sevilla, Seville, Spain; 17 Institute of Laboratory Medicine, University Hospital, LMU Munich, Munich, Germany; 18 Department of Child and Adolescent Psychiatry and Psychology, Institute of Neurosciences, Hospital Clinic Universitari, Barcelona, Spain; 19 Institut d’Investigacions Biomediques August Pi i Sunyer (IDIBAPS), Barcelona, Spain; 20 Centro de Investigacion en Red de Salud Mental (CIBERSAM), Instituto Carlos III, Madrid, Spain; 21 Department of Psychiatry and Psychotherapy, University Hospital, LMU Munich, Munich, Germany; 22 Marion von Tessin Memory-Zentrum gGmbH, Munich, Germany; 23 Clinic of Psychiatry, Social Psychiatry and Psychotherapy, Hannover Medical School, Hannover, Germany; 24 Institute of Neurogenetics, University of Lübeck, Lübeck, Germany; 25 Child and Adolescent Mental Health Center, Mental Health Services, Capital Region of Denmark and University of Copenhagen, Copenhagen, Denmark; 26 Service of Child and Adolescent Psychiatry, Department of Psychiatry, University Medical Center, University of Lausanne, Lausanne, Switzerland; 27 Azienda Sanitaria Locale di Bari, Mental Health Department, Child and Adolescent Neuropsychiatry Service of Bari Metropolitan Area, Bari, Italy; 28 Department of Biological Sciences, Purdue University, West Lafayette, USA; 29 Child Neuropsychiatry Section, Department of Clinical and Experimental Medicine, School of Medicine, Catania University, Catania, Italy; 30 Department of Child and Adolescent Psychiatry, Faculty of Medicine, TU Dresden, Dresden, Germany; 31 Vadaskert Child and Adolescent Psychiatric Hospital, Budapest, Hungary; 32 Department of Child and Adolescent Psychiatry, University Medical Center Groningen, University of Groningen, Hanzeplein 1, 9713 GZ Groningen, The Netherlands

**Keywords:** Genetics, Longitudinal, Obsessive–compulsive disorder, Stress, Streptococcal infection, Tourette syndrome

## Abstract

Genetic predisposition, autoimmunity and environmental factors [e.g. pre- and perinatal difficulties, Group A Streptococcal (GAS) and other infections, stress-inducing events] might interact to create a neurobiological vulnerability to the development of tics and associated behaviours. However, the existing evidence for this relies primarily on small prospective or larger retrospective population-based studies, and is therefore still inconclusive. This article describes the design and methodology of the EMTICS study, a longitudinal observational European multicentre study involving 16 clinical centres, with the following objectives: (1) to investigate the association of environmental factors (GAS exposure and psychosocial stress, primarily) with the onset and course of tics and/or obsessive–compulsive symptoms through the prospective observation of at-risk individuals (ONSET cohort: 260 children aged 3–10 years who are tic-free at study entry and have a first-degree relative with a chronic tic disorder) and affected individuals (COURSE cohort: 715 youth aged 3–16 years with a tic disorder); (2) to characterise the immune response to microbial antigens and the host’s immune response regulation in association with onset and exacerbations of tics; (3) to increase knowledge of the human gene pathways influencing the pathogenesis of tic disorders; and (4) to develop prediction models for the risk of onset and exacerbations of tic disorders. The EMTICS study is, to our knowledge, the largest prospective cohort assessment of the contribution of different genetic and environmental factors to the risk of developing tics in putatively predisposed individuals and to the risk of exacerbating tics in young individuals with chronic tic disorders.

**Electronic supplementary material:**

The online version of this article (10.1007/s00787-018-1190-4) contains supplementary material, which is available to authorized users.

## Introduction

Tic disorders are common, childhood-onset neuropsychiatric conditions characterised by the presence of sudden, rapid, recurrent, non-rhythmic motor movements (motor tics) and/or vocalisations (vocal tics). Tic disorders are diagnosed when motor tics and vocal tics, either alone (chronic motor or vocal tic disorder) or in combination [Tourette syndrome (TS), manifesting with multiple motor tics and at least one vocal tic], begin before age 18 and last more than 1 year, in the absence of tics being attributable to a substance or another medical condition [1]. The prevalence of tics during childhood/adolescence is close to 3%, and that of TS is approximately 0.8% between the ages of 6 and 18 years [2–4]. Tics and their associated neuropsychiatric comorbidities [attention-deficit/hyperactivity disorder (ADHD), obsessive–compulsive disorder (OCD), anxiety and depressive disorders, autism spectrum disorders] often affect quality of life of patients and families, as well as social and academic functioning of patients [5–9].

Our knowledge of the pathophysiological mechanisms involved in TS and other chronic tic disorders is still limited. Pharmacological and behavioural treatment options for TS represent the mainstay of treatment for tics, but both have limitations related to patients’ access to care, tolerability, and efficacy. In addition, at least 5% of patients with TS attending specialized clinics may have a very severe form that is refractory to non-invasive treatments [10]. There is still major need for new treatments and effective preventative methods, potentially fostered by a better understanding of disease pathophysiology.

TS is viewed as a complex neuropsychiatric disorder, which is likely to be related to an as yet poorly understood interaction between genetic and environmental susceptibility factors. While the heritability of TS has been estimated to be as high as 0.77 in a large scale multigenerational family study [11], a recent twin-family study found much lower heritability estimates, ranging between 0.25 and 0.37 [12], indicating a substantial role for environmental factors. The complex trait of tic disorders is polygenic, similar to most psychiatric disorders. Over the past decade, genetic factors associated with TS have been explored primarily through genome-wide approaches including genome-wide association studies (GWASs), analysis of copy number variants (CNVs), and whole exome sequencing (WES) approaches. GWASs in TS have to date failed to identify highly genome-wide significant loci [13, 14], likely due also to limited sample sizes, which were smaller than in other major psychiatric GWASs [15]. The contribution of rare structural variation to the genetic architecture of TS is supported by recent analyses of rare CNVs, which indicate that approximately 1% of TS cases carry one of these CNVs, highlighting also genome-wide significant loci increasing TS risk, i.e. *NRXN1* deletions and *CNTN6* duplications [16]. Like GWASs, WES studies in TS are also limited by their small sample sizes compared to other complex psychiatric traits; an association with de novo damaging variants has been reported for a dozen candidate genes [17–20] and needs to be confirmed by studies with larger sample sizes. There is also a striking paucity of gene expression studies in tic disorders. Studies in this area focused on biological pathways related to neurotransmitters and immune regulation [17, 21–23], but were based on small sample sizes, did not clarify whether the observed changes were causes or consequences of the behavioural phenotype, and were never adequately combined to genomic data. Finally, the exploration of epigenetic modifications associated with TS and other chronic tic disorders is still in its dawning [12, 24].

Some pre- and perinatal factors potentially interfering with normal brain development have been explored also in association with TS, although the related evidence differs for quality and methodology used across the different variables explored [e.g. 25–29]. During the past decade, a limited number of studies have also explored the contribution of psychosocial stress with overall inconclusive results, even if clinical experience does seem to support a role of stress in patients with TS [30]. A prospective evaluation based on questionnaires suggested an effect of psychosocial stress as a short-term predictor of tic severity [31]. Another study showed increased cortisol responses during acute stressors [32], but this was not confirmed by Buse et al. [33], who observed the opposite acute effect on a behavioural acute stress test (Trier Social Stress Test), coupled to salivary cortisol determination and autonomic measures of the stress response. Obvious limitations of this literature are the limited sample sizes and the lack of longitudinal data exploring biological markers of acute and chronic stress, as well as direct measures of hypothalamic–pituitary–adrenal (HPA) axis activation.

An important research area, relevant to environmental influences in relation to tic disorders, is the involvement of abnormal innate and adaptive immune responses in the pathogenesis of tics and related behavioural symptoms [34]. A dysfunctional neural-immune cross-talk has been observed in patients with TS, in analogy to other neurodevelopmental disorders (e.g. autism spectrum disorder). Recent data from prospective population-based cohorts have demonstrated a 30% increased incidence rate of TS in male offspring of women with an autoimmune disease [34], and a higher risk of any autoimmune disease among first-degree relatives of patients with OCD and chronic tic disorders [35]. Clinical studies have documented increased proliferation and activation of B and T_H1_ lymphocytes, increased pro-inflammatory cytokine levels, a decreased number of *T*_REG_ lymphocytes, dysregulated immunoglobulin synthesis, supporting the existence of adaptive immune responses skewed towards an inflammatory state TS [36, 37]. Furthermore, both post mortem data [38] and in vivo molecular imaging [39] suggest microglial activation in TS. Microglia cells are key players of the immune system in the central nervous system and play an increasingly recognized role in brain infections, neuroinflammation and neurodegeneration [40].

Alongside stress, infectious pathogens are obvious potential culprits for the overactivity of immune responses documented in tic disorders. A specific interest in a role for common infections (pharyngotonsillitis) caused by group A streptococcus (GAS or *Streptococcus pyogenes*) has been drawn by the description in 1998 of Paediatric Autoimmune Neuropsychiatric Disorders Associated with Streptococcal Infections (PANDAS), a putatively autoimmune syndrome manifesting with obsessive–compulsive symptoms, tics, emotional lability, anxiety and regressive behaviour triggered by this pathogen [41, 42]. In 2012, the broader syndromic entity of Pediatric Acute Neuropsychiatric Syndromes (PANS) has been proposed, which encompasses PANDAS but includes other possible aetiologies [43]. Both PANS and PANDAS are viewed as a subtype of paediatric OCD and/or TS that present with an abrupt onset or exacerbation of neuropsychiatric symptoms [44]. An autoimmune mechanism triggered by molecular mimicry between GAS bacterial and host neural antigens has been proposed for PANDAS, and putatively pathogenic biomarkers, e.g. autoantibodies directed against dopamine D2 receptors and antibody-mediated calcium/calmodulin-kinase II activation in cell-based assays, have been reported and included in a proposed diagnostic panel (the Cunningham panel) [45, 46]. The diagnostic accuracy of this panel is, however, still discussed [47], and the potential usefulness of these biomarkers in highlighting an ‘autoimmune’ subgroup of patients with tic disorders is unexplored. More recent work has highlighted the rationale of exploring immune regulatory markers in this group of patients, and decreased systemic levels of vitamin D have been documented in an Italian cohort of patients with PANDAS [48]. Preliminary evidence reported the presence of anti-D2 dopamine receptor antibodies in a small proportion of individuals with TS Dale [49], supporting the rationale for further exploration of autoantibody markers in this condition.

Although tics are now considered an associated rather than a core feature of PANDAS and PANS [42], researchers have tried to address, during the past decade, the still unanswered question of whether and how infections, and above all GAS infections, are associated with the onset and/or the exacerbation of tics and related behavioural symptoms. Large, retrospective, population-based cohort studies have provided some degree of evidence that GAS infections may be associated with the onset of tics [50–53] although discrepancies across studies exist [54]. At the same time, smaller, prospective studies have failed to show a clear association between GAS infections and tic exacerbations [55–59]. Nevertheless, one of these prospective studies reported a multiplicative interaction between GAS infections and psychosocial stress in predicting tic and obsessive–compulsive symptom severity in the short term [60]. Overall, these previous studies are limited by small sample sizes and some ambiguities in design (addressed in more depth in our “Discussion”), which justify the conduction of larger, more ambitious, prospective observations that take into account the complexity of the clinical phenomenology of tic disorders, the genetic diversity of GAS, and the high inter-individual variability of GAS-induced immune responses [61]. An adequately sized clinic-based prospective study that collects behavioural, microbiological, immune-endocrinological, genomic and transcriptomic data, provides a unique opportunity to advance the field by tackling some of the unanswered questions on the aetiopathogenesis of chronic tic disorders. The objectives and design of the multi-centre pan-European collaborative study EMTICS, funded by the European Commission seventh Framework programme (FP7), were conceived to address several of the knowledge gaps summarized above.

## Core objectives of EMTICS

1. To investigate the association between putatively relevant environmental factors, genome-wide genetic factors, and gene expression patterns upon the risk of developing clinically relevant exacerbations of tics and/or OCD symptoms in youth with an established chronic tic disorder (COURSE cohort).
2. To investigate the association between putatively relevant environmental factors, genome-wide genetic factors, and gene expression patterns upon the risk of new onset of tics in children who are first-degree relatives of patients with an established chronic tic disorder (ONSET cohort).

For the first and second objectives, the explored environmental exposures comprise: recent GAS infection, GAS carriage status, other recent infections, fluctuations of psychosocial stress, cortisol as a marker of chronic stress and pre- and perinatal adversities.

3. To characterise patterns of the host innate and adaptive immune responses that are associated with clinical events of interest, i.e. onset of tics and clinically relevant exacerbations of tics and/or OCD symptoms in the two clinical cohorts. This will comprise analyses of immune effectors (e.g. cytokines, immunoglobulins, acute phase reactants, other effector molecules including those belonging to the tryptophan/kynurenine pathway and vitamin D) and immune cell phenotyping.
4. To characterise patterns of the host antibody response to GAS and other pathogens previously reported in association with chronic tic disorders. The anti-GAS antibody patterns will be investigated with state-of-the-art microarray technology.
5. To develop multimodal prediction models for the risk of onset of tics in first-degree relatives of patients with chronic tic disorders, as well as for the risk of clinically relevant exacerbations of tics and/or OCD symptoms in youth with an established chronic tic disorder.

## Methods

### Participants

Children aged 3–16 years were recruited from the sixteen child and adolescent psychiatry and paediatric neurology outpatient clinics (listed in the “Appendix”), from patients and families already known to these services or through advertisement of the study to patient organizations and other health professionals. This project was based on two separate cohort studies: ONSET and COURSE. The study was approved by the Institutional Review Boards of the participating centres. Parents and their child (ren) provided written informed consent and assent as appropriate according to ethical regulations. While travel costs of participating families were reimbursed no additional fees were paid to participants. The first participant was enrolled in EMTICS in January 2013 and the study has concluded with the last visit of the last ONSET patients in June 2018.

#### Inclusion criteria

For the ONSET cohort, children aged 3–10 years were recruited who are first-degree relatives (siblings or children) of patients with TS or another chronic tic disorder (criteria according to the Diagnostic and Statistical Manual fourth edition, text revision [62]), but had themselves never had tics, OCD or trichotillomania. This age range reflects the range of age at first onset of tics in chronic tic disorders, as reported by the majority of naturalistic studies on these conditions [63]. For the COURSE cohort, patients aged 3–16 years with an established diagnosis of TS or chronic motor or vocal tic disorder according to DSM IV-TR criteria [62] were recruited. More than one sibling per family could participate in both studies.

#### Exclusion criteria

Children unable to understand and comply with protocol, or whose parents were unable to comply, and children with serious medical/neurological illness or treated with antibiotics in the past month were excluded from both ONSET and COURSE cohorts. The presence of comorbid neuropsychiatric conditions such as autism spectrum disorder was not an exclusion criterion, nor was suspected PANS or PANDAS. Use of medications was not an exclusion criterion; however, all medication a child was taking during the study was recorded. Children who refused throat swabs, blood draws or hair strains could still participate in the studies.

### Study design and procedures

#### Longitudinal study ONSET

**Fig. 1:**
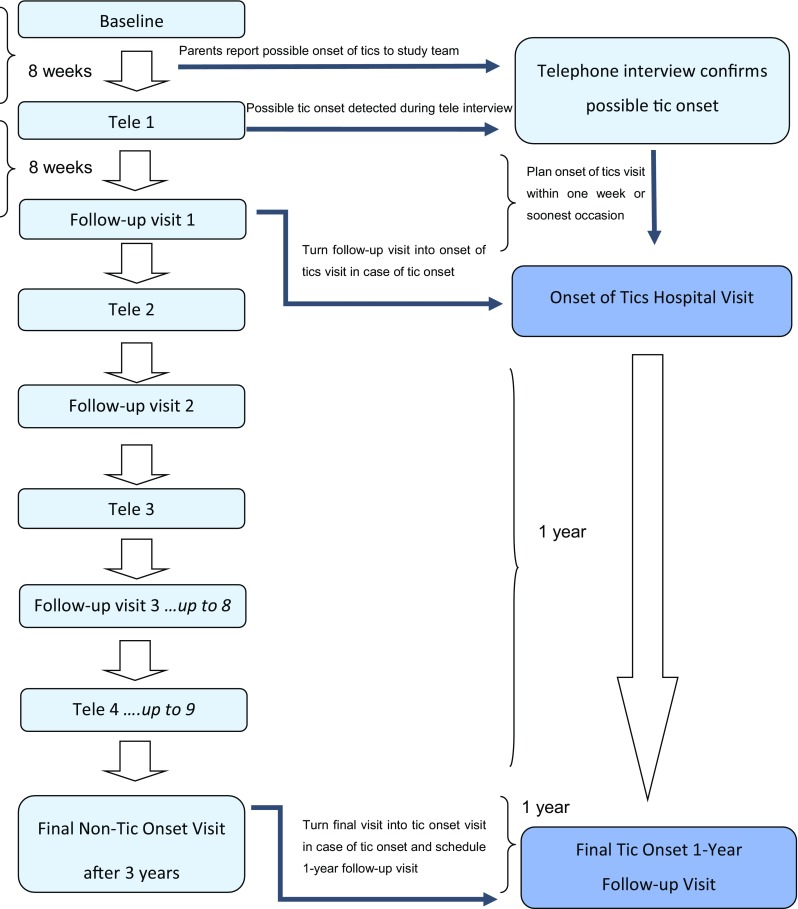
ONSET study flow chart. *Tele* telephone interview. If no tic onset was detected and confirmed then the original assessment schedule (left side) was retained. After an onset of tics visit, all assessments were discarded, except for a 1-year follow-up visit. The minimum study period was 1 year, tic onset at the final visit increased the maximum study period to 4 years

The ONSET study examines the association of the new onset of tics with exposure to pharyngeal GAS carriage or infection and other environmental and genetic factors, using clinical and laboratory assessments, over a 3-year period where the onset of tics represents the event of interest. Participants were evaluated every 2 months, interchangeably by 4-monthly scheduled hospital visits and telephone interviews. Telephone interviews were scheduled in the middle of the 4 months’ intervals between hospital visits (see Fig. 1 and supplementary Table 1). Irrespective of the planned visit schedule, parents were instructed to communicate any possible sign of tic onset to the study centre as soon as possible (e.g. by phone or email). To this purpose, all possible symptoms indicative of a possible onset of tics were thoroughly explained to parents at the baseline visit in ONSET.

Data collection was structured on three levels of observation: (1) through a weekly diary in which children’s parents were asked to indicate symptom onset, aimed at the earliest possible detection of onset of tics throughout the whole study duration plus an inventory of signs of infectious disease (common cold), obsessive–compulsive symptoms and stressful events; (2) scheduled telephone interview once every 4 months with review of the weekly diaries since the last assessment and clinical evaluations performed by the study clinician to children’s parents; and (3) visits in hospital every 4 months over the 3-year duration of the follow-up period, which comprised a more extensive clinical evaluation and collection of biological specimens (for measurements see Table 1).

**Table 1:** Summary of clinical and laboratory measurements at ONSET visits

	Baseline visit	4-monthly follow-up visit	Tic onset visit	Final visit 1-year post-tic onset^a^	Final visit without tic onset
Demographics and family medical history	✓				
Child’s medical history	✓			✓ (update)	✓ (update)
Inventory of infections	✓	✓^b^	✓		✓
Psychotropic drug checklist	✓	✓	✓	✓	✓
Prenatal, perinatal and developmental history	✓				
Exploration of possible tic onset		✓^b^			✓
YGTSS			✓	✓	
PUTS			✓	✓	
CY-BOCS	✓	✓	✓	✓	✓
CGI-S (tics)			✓	✓	
CGI-S (overall)	✓	✓	✓	✓	✓
CGI-I (overall)		✓	✓	✓	✓
Tic disorder diagnosis				✓	
OCD diagnosis				✓	✓
ADHD diagnosis	✓			✓	✓
Trichotillomania diagnosis				✓	✓
SDQ	✓	✓	✓	✓	✓
SNAP-IV	✓	✓	✓	✓	✓
ASSQ	✓				
Kindl-R	✓	✓	✓	✓	✓
PSS-P-10; PSS-C-10 (≥ 11 years)	✓^c^	✓^d^	✓		✓
Inventory of stressful events	✓	✓^b^	✓		✓
Throat swab	✓	✓^e^	✓		✓
Serum sample for immune analyses^f^	✓	✓^g^	✓		✓
Fresh blood sample^h^	✓		✓		
Blood sample for DNA			✓		
Blood sample for RNA	✓		✓		
Hair sample for cortisol analysis	✓	✓	✓		✓

See the main text for a description of measures

^a^The purpose of this visit was to establish whether the onset of tics was indeed indicative of the onset of a chronic tic disorder and to assess the possible presence of comorbidity

^b^Also assessed during 4-monthly telephone interviews

^c^No PSS-C-10

^d^PSS-P-4 during 4-monthly telephone interviews

^e^Only for follow-up visits #3 and #6

^f^Antibody responses to Group A streptococcal infections and other infectious pathogens including Anti-streptolysin O (ASO) and anti-deoxyribonuclease B antibody titres (anti-DNAse B), cytokines, inflammatory status (C-reactive protein), and autoantibodies

^g^ASO and anti-DNAse B measurements only

^h^For immune response analyses only in a sub-sample of participants

If parents reported possible onset of tics outside of planned visits or telephone interviews, an “unscheduled tic onset evaluation telephone interview” was held by the study clinician to investigate whether possible onset of tics had occurred. Tic onset was defined as the first occurrence of any sudden, rapid, recurrent, non-rhythmic involuntary motor movement and/or vocalisation noticed on at least three separate days within a period of 3 weeks. If the evaluation pointed to a possible tic onset (even when somewhat unsure), an “onset of tics hospital visit” was scheduled preferably within 1 week or at the earliest opportunity for extended clinical evaluation and collection of biological material. This served to assess the range of tics and/or obsessive–compulsive symptoms, symptoms and signs of infection (through throat swabs and blood tests for immunological markers of infection).

An “onset of tics hospital visit” was also scheduled when a (not previously reported) possible onset of tics was detected during a scheduled telephone interview while reviewing parents’ weekly diaries over the past 2 months since the last assessment. Likewise, a planned follow-up hospital visit was turned into an “onset of tics hospital visit” when a possible onset of tics was confirmed during the planned visit. If tic onset occurred, all further planned assessments were discarded whereas a final follow-up visit was scheduled 1 year later that consisted of a clinical evaluation to confirm the true onset and type of tic disorder (according to DSM-IV-TR criteria) [62]. If an onset of tics was definitely not confirmed (e.g. tic-like phenomena could be better explained as symptoms of allergy or infectious disease), then the original visit schedule was retained. The maximum duration of the follow-up period was 3 years from enrolment if there was no tic onset (concluding with a “final non-tic onset visit”; a maximum of ten planned hospital visits and nine telephone interviews took place; see supplemental Table 1), and a maximum of 4 years from enrolment if tics developed (concluding with a “final tic onset 1-year follow-up visit”, see Fig. 1).

#### Longitudinal study COURSE

**Fig. 2:**
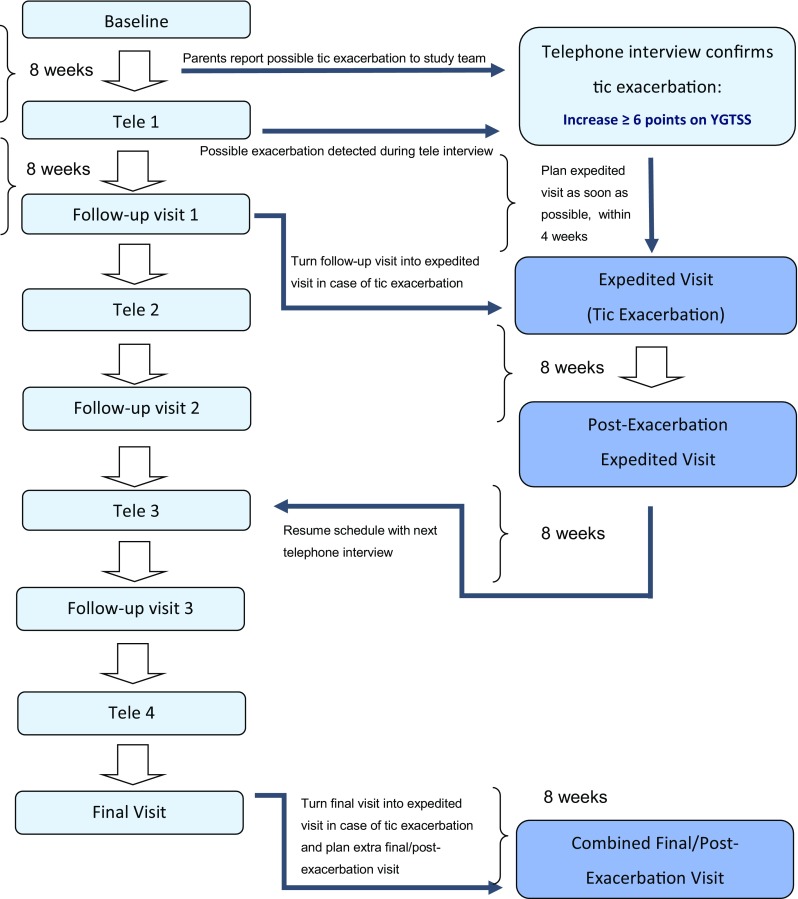
COURSE study flow chart. *Tele* telephone interview, *YGTSS* Yale Global Tic Severity Scale. If no tic exacerbation was detected and confirmed then the original 16 month assessment schedule (left side) was retained. The maximum study period was 18 months if a tic exacerbation was detected at the final visit. A maximum of two pairs of expedited and post-exacerbation visits was possible. Expedited visits shortened the study period accordingly

The COURSE study is a cohort study that prospectively observed over a maximum period of 18 months 715 children and adolescents with an age range of 3–16 years affected by a tic disorder [62], where exacerbations of tics represent the events of interest. Data collection was analogous to the ONSET study, with interchanging 4-monthly planned hospital visits and telephone interviews as well as parental weekly diaries aimed at identifying tic exacerbations (see Fig. 2 and supplementary Tables 2 and 3).

Similar to the ONSET protocol, parents were asked to report any noticeable increase in tic severity, seemingly unrelated to a reduction or discontinuation of prescribed medication, to the study team. If the study clinician subsequently through an unscheduled telephone interview confirmed a tic exacerbation, defined as an increase of at least six points on the total tic severity score (i.e. sum of motor and vocal tic severity, but not impairment) of the Yale Global tic severity scale (YGTSS) [64] compared to the score during the previous assessment, an appointment for an expedited visit was scheduled preferably within 1 week, but no more than within 4 weeks. This expedited visit was followed by a “post-exacerbation visit” 2 months later, aimed at capturing tic remission, defined as a subsequent decrease of at least six points on the total tic severity score. Thereafter, a bi-monthly schedule of either a telephone interview or hospital visit was resumed. In COURSE, the total number of hospital visits was five with four planned telephone interviews, or six visits when an exacerbation had occurred at the final visit; a maximum number of two pairs of exacerbation plus post-exacerbation visits were possible (see supplementary Table 3 for the different measurement scenarios).

An exacerbation of tics (not previously reported by parents) may also have been detected during the bi-monthly assessments either through telephone or hospital visit while reviewing the weekly diaries. While preferably the exacerbation should then still be ongoing it was allowed to schedule an expedited visit within 4 weeks of the tic exacerbation. Thus, it may have occurred that tic severity was already in remission at the time of the expedited visit. If a tic exacerbation came under the attention during a planned follow-up visit, then the visit was turned into an “expedited tic exacerbation visit”. Finally, in some of the participating clinical sites, COURSE participants were considered for inclusion in a separate antibiotic treatment study if their microbiological testing revealed a positive culture for GAS.

#### Missed visits and end of study

Planned follow-up hospital visits and telephone interviews as well as “post-exacerbation visits” could be scheduled 2 weeks earlier or later than according to the visit schedule (see supplementary Tables 1 and 2; except “tic onset visits” that could be scheduled at the earliest opportunity, or “expedited tic exacerbation visits” and “final visits” that could be scheduled with a delay of up to 4 weeks). If this was not possible, the visit or interview was considered missed. Three consecutive missed appointments (visits or telephone interviews) led to study discontinuation. Children not keeping weekly diaries could still continue to participate in the study.

Study end is the date of the last visit of the last participant; for the ONSET study this is after 3 years after baseline in case of no tic onset; or 1 year after onset of tics with a maximum of 4 years. For the COURSE study, this is after 16 months or less if there were tic exacerbations, and a maximum of 18 months when a tic exacerbation was identified during the planned final visit.

### Clinical and laboratory measures

#### Clinical measures

Tables 1, 2, 3 present a summary of clinical and laboratory measurements at ONSET and COURSE visits, respectively. The main outcome measure for both cohort studies is based on the YGTSS, the gold standard instrument to rate tic severity, which has robust psychometric properties [64]. Another main, well-validated measure was the Children’s Yale Brown Obsessive–Compulsive Scale (CY-BOCS; [65]), used to rate obsessive–compulsive symptom severity. Study clinicians well-experienced in the evaluation and treatment of tic disorders and associated conditions used these semi-structured interviews to assess past week symptom severity and the current and past presence of tic disorders, OCD, and attention-deficit/hyperactivity disorder (ADHD) according to DSM-IV-TR criteria [62]. Clinicians also rated the Clinical Global Impression Scale Severity for overall functioning and/or tics (CGI-S for severity and CGI-I for improvement) [66] and checked a list of psychotropic medication use during the past 2 weeks.

**Table 2:** Summary of clinical and laboratory measurements at COURSE visits

	Baseline visit	4-monthly follow-up visit	Expedited visit in case of tic exacerbation	Post-exacerbation visit^a^	Final visit
Demographics and family medical history	✓				
Child’s medical history	✓				✓ (update)
Inventory of infections	✓	✓^b^	✓	✓	✓
Psychotropic drug checklist	✓	✓	✓	✓	✓
Prenatal, perinatal and developmental history	✓				
Evaluation of possible tic exacerbation		✓^b^			✓
YGTSS	✓	✓^b^	✓	✓	✓
PUTS	✓	✓	✓	✓	✓
CY-BOCS	✓	✓^b^	✓	✓	✓
CGI-S (overall and tics)	✓	✓	✓	✓	✓
CGI-I (overall and tics)		✓	✓	✓	✓
Tic disorder diagnosis	✓				✓
OCD diagnosis	✓				✓
ADHD diagnosis	✓				✓
Trichotillomania diagnosis	✓				✓
SDQ	✓	✓	✓	✓	✓
SNAP-IV	✓	✓	✓	✓	✓
ASSQ	✓				
Kindl-R	✓	✓	✓	✓	✓
PSS-P-10; PSS-C-10 (≥ 11 years)	✓	✓^c^	✓	✓	✓
Inventory of stressful events	✓	✓^b^	✓	✓	✓
Throat swab	✓	✓	✓	✓	✓
Serum sample for immune analyses^d^	✓	✓^e^	✓	✓	✓
Fresh blood sample^f^			✓	✓	
Blood sample for DNA	✓				
Blood sample for RNA			✓	✓	
Hair sample for cortisol analysis	✓	✓	✓	✓	✓

See the main text for a description of measures

^a^Two months after the expedited visit to capture possible tic remission

^b^Also assessed during 4-monthly telephone interviews

^c^PSS-P-4 during 4-monthly telephone interviews

^d^Antibody responses to Group A streptococcal infections and other infectious pathogens including anti-streptolysin O (ASO) and anti-deoxyribonuclease B antibody titres (anti-DNAse B), cytokines, inflammatory status (C-reactive proteins), and autoantibodies

^e^ASO and anti-DNAse B measurements only

^f^For immune response analyses only in a sub-sample of participants

**Table 3:** Laboratory measures

Analysis	Material	Laboratory parameters and laboratories
GAS colonisation	Throat swabs	Bacterial group A streptococcal (GAS) population at each individual centre
Anti-streptococcal antibody titres	Serum	Anti-streptolysin O (ASO), Anti-desoxyribonuclease B (anti-DNAseB) at LMU
Antibodies to non-streptococcal pathogens	Serum	Mycoplasma pneumoniae (Myco_IgG), Chlamydia trachomatis (Chlamy_IgG), Epstein–Barr virus (EBV), Borrelia burgdorferi (Borrel_IgG), and Toxoplasma gondii (Toxo_IgG) at LMU
Anti-streptococcal immune response	Serum	Antibody responses to GAS multiple antigens at GSK
Autoantibodies by cell-based assay	Serum	Anti-neuronal antibodies targeting candidate self-antigens for post-streptococcal neuropsychiatric disorders, mainly dopamine D2 receptors at Bari
Cytokine receptors and immunoglobulins	Serum	Interleukin-6 (IL-6), interleukin-17F (IL-17F), tumor necrosis factor α (TNF-α), TNF-RI, TNF-RII, CD14, immunoglobulin subclasses (IgA, IgM, IgG1-4) at Cytolab
C-Reactive Protein (CRP)	Serum	Pentameric CRP (pCRP), Monomeric CRP (mCRP) at ApDia
Tryptophan and kynurenine pathway intermediates	Serum	Tryptophan (TRP), kynurenine (KYN), kynurenic acid (KYNA), 3-hydroxykynurenine (3HK), xanthurenic acid (XAN), anthranilic acid (AA), quinolinic acid (QUIN), picolinic acid (PIC), 5-hydroxytryptophan (5-HTRP), 5-hydroxyindoleacetic acid (5HIAA), nicotinic acid (NAD) at LMU
Vitamin D	Serum	25-OH-Vitamin D at LMU
T cells and NK cells	Whole blood	IFN-g, CD4/CD8, CD56/CD3 at ProImmune
Genotyping and gene expression	PAXgene tubes (RNA), EDTA tubes (DNA)	Genome-wide genetic factors, genotyping and gene expression at deCODE Genetics and Biolytix
Hair cortisol	Hair strains 2–4 cm	Cortisol measuring chronic stress in hair at TUD

See the “Appendix” for laboratory centres

A set of parent-reported questionnaires was used to assess participants’ demographics, the child’s medical history (e.g. immune-related conditions such as allergic rhinitis or atopic dermatitis), family medical history (psychiatric, neurologic and autoimmune diseases) [66], prenatal, perinatal and developmental history (Modified Schedule for Risk and Protective factors Early in Development, MSRPFED) capturing 38 possible adverse situations [28, 67, 68] and degree of child experienced psychosocial stress over the previous month by the Perceived Stress Scale (PSS-P-10 at hospital visits; PSS-P-4 in telephone interviews) [69]. Parent ratings of child’s psychopathological symptoms included the Strengths and Difficulties Questionnaire (SDQ) [70, 71], the Swanson, Nolan and Pelham-version IV rating scale (SNAP-IV) [72, 73] to assess ADHD severity, and the Autism Spectrum Screening Questionnaire (ASSQ) [74]. Finally, the Kindl-R questionnaire (4–7 and 8–16 years parent versions) [75] measured health-related impact on the quality of life.

Child reports were the Premonitory Urge for Tics Scale (PUTS) [76], which measures unpleasant sensorimotor phenomena that often precede tics and the self-reported Perceived Stress Scale (PSS-C-10) [69]) in children ≥ 11 years. The weekly diaries covered an inventory of possible infections (i.e. symptoms of common cold such as running nose, persistent cough or fever) and related diagnosis and treatment, possible medication changes, occurrence of stressful events, and possible onset of tics or fluctuations (especially exacerbations) in tics and OCD symptoms.

#### Laboratory measures

See Table 3 for an overview of laboratory measures. The main microbiological measures were GAS colonisation by throat swabbing and processing using a consensus-defined methodology (pour plate method) [55, 77]. The microbiological typing of bacterial GAS population was performed by emm typing, Multiple Locus Variable number of tandem repeats Analysis (MLVA) and Multi Locus Sequence Typing (MLST). Exposure to GAS in study participants was investigated by measuring anti-streptolysin O titre (ASOT) and anti-DNAseB antibody titre. Moreover, protein microarray technology described elsewhere by one of the teams participating in the EMTICS consortium [61] was used to compare the patterns of antibody responses to a panel of GAS antigens in ONSET participants who have or have not developed tics. By the same approach, antibody profiles in the COURSE participants were compared pre and post exacerbation.

Other performed serum immune measurements included measurement of IgA-, IgM- and IgG-titres against *Mycoplasma pneumoniae*, *Chlamydia* spp., Epstein–Barr virus (EBV), *Borrelia burgdorferi*, and *Toxoplasma gondii*; sCD14, interleukin-6, interleukin-17, tumor necrosis factor (TNF)-α, TNF-RI, TNF-RII, immunoglobulin (IgA, IgM, IgG) subclasses, and ratio between pentameric and monomeric CRP to examine the possibility of impaired resolution of inflammation; tryptophan, kynurenine, kynurenic acid, 3-hydroxykynurenine, xanthurenic acid, anthranilic acid, 3-hydroxyanthranilic acid, quinolinic acid, picolinic acid, 5-hydroxyindoleacetic acid and other important intermediates of the tryptophan pathway. Furthermore, the vitamin D status was measured and in a small sub-sample, immune cell-based measures include phenotyping for the main innate and adaptive immunological cell types, including T cells (CD3, CD4, CD8), B cells (CD19) and natural killer cells (CD56+). Functional analysis assessed the production of inflammatory cytokines post-stimulation/activation by bacterial pathogen triggers. Autoantibodies measured in serum, by cell-based assay [49] included anti-neuronal antibodies targeting candidate self-antigens for post-streptococcal neuropsychiatric disorders, mainly dopamine D2 receptors, in relation to onset or exacerbation of tic disorders.

Genome-wide genetic factors were investigated through the genotyping of participants using peripheral blood-extracted DNA, genotyped on an Illumina Human OmniExpress array, targeting more than 700,000 common variants. Published genome-wide genotyping data for individuals of European descent are used for the control dataset to perform GWAS and identify single gene or gene–gene interactions associated with TS and obsessive–compulsive symptoms. The integration of EMTICS genetics data with existing datasets from parallel efforts from European and American consortia will enable a meta-analysis of a joint dataset with more than 8000 cases with TS and obsessive–compulsive symptoms. In addition, genome-wide gene expression data were investigated using peripheral blood-extracted total RNA, analysed on an Affymetrix Human Transcriptome Array (HTA 2.0), targeting more than 285,000 coding and non-coding and alternatively spliced transcripts to unravel biological pathways that may influence the onset and clinical course of tics activated upon symptom exacerbations and remissions. By combining genotyping and gene expression data with other laboratory measures, we aim to explore the complex interaction between the environmental, immunological and autoimmune factors related to the onset and clinical course of the disorder spectrum. Finally, we determined cortisol levels in hair, which is a biomarker of retrospective chronic stress [78] using a commercially available immunoassay with chemoluminescence detection (CLIA, IBL-Hamburg, Germany) [79, 80].

### Sample size calculations

#### ONSET study

The ONSET study originally aimed to recruit 500 participants but managed to include 260 children who are first-degree relatives of patients with a tic disorder, aged 3–10 years. Still this sample size would allow us to obtain an estimated odds ratio of 2.85 for GAS carriers compared to non-carriers with respect to the event “onset”, using the pre-study assumptions of an estimated GAS carriage rate during childhood of 15% [81], and an estimated risk of 30% [82] for a first-degree relative of a patient with TS or another chronic tic disorder to be affected by a tic disorder or OCD. Assuming a 15% GAS carriage rate and 30% risk of TS/OCD in siblings or children of patients with TS at age 3–10, to obtain an odds ratio of 2.2 for GAS carriers compared to non-carriers with respect to the event “onset” with 80% power and 5% significance level (two-sided), we calculated a sample size of 319 using the Kelsey method. Assuming a 15% drop out a sample size of 375 patients allows this number to be achieved.

#### COURSE study

The COURSE study includes 715 children and adolescents affected by a tic disorder with an age range of 3–16 years. A target sample size of 700 was based on the following pre-study assumptions: we assumed it would allow us to obtain an estimated odds ratio of 2.45 for GAS carriers compared to non-carriers with respect to the event “exacerbation”, assuming an exposure to streptococcal infection of 0.12 and a rate of symptom exacerbation of 0.16 based on a conservative estimate from prior longitudinal studies of TS [58, 59]. The sample size for the COURSE study was calculated in an identical fashion to the ONSET study.

#### Genetic and gene expression analyses

The sample collected in this study will provide 96% power to detect genetic alleles of frequency > 0.3 conferring risk > 1.5, to be analysed using existing tools (e.g. methods implemented in PLINK), as well as by designing novel statistical and data mining techniques, aiming to uncover single gene, gene–gene and gene–environment interaction in TS/OCD pathogenesis. For gene expression group analysis, for a minimum total of 40 samples (20 samples per group), we can reach significant results (*p* < 0.05/16,000 = 3 × 10^−6^) with 98% power if considering about 16,000 genes remaining after quality control, with the gene(s) showing foldChange ≥ 2 (up/down regulation).

### Statistical analysis

#### ONSET study

We will use logistic regression to evaluate risk factors for developing tics, while survival analysis (Cox proportional hazards model) will be performed to assess the effect of risk factors on the time of developing tics, allowing for clustering of patients within regions. We will also develop a risk model for the risk of new onset of tics in first-degree relatives of patients with chronic tic disorders. Cox proportional hazards regression models (with adjustments for clustering) will be used to develop the risk model. Selection or shrinkage methods may be employed depending on the event rate.

#### COURSE study

Data analysis of the COURSE study will be directed at examining the association between the longitudinal course of tics and/or obsessive–compulsive symptoms and various risk factors. Logistic regression will be used to evaluate the effects of risk factors on the risk of tic exacerbation during the same or the subsequent follow-up period, while survival analysis (Cox proportional hazards model) will be performed to assess the effect of risk factors on the time to development of exacerbations, again allowing for clustering of patients within regions.

In both studies, exposure to GAS will be defined as new GAS throat carriage as evaluated on microbiological analysis of the throat specimen (‘newly positive’ throat swab) OR as significant elevation of anti-streptococcal antibody titres, defined as follows: a significant elevation of anti-streptolysin O titre (ASOT) will be diagnosed when ASO > 200 AND [log10 (ASOcurrent visit) − log10(ASOprior visit)] ≥ 0.2 (variation between log10 for two consecutive measurements is higher than or equal to 0.2); a significant elevation of anti-DNAseB will be diagnosed when anti-DNAseB > 300 AND [log10 (anti-DNAseBcurrent visit) − log10(anti-DNAseBprior visit)] ≥ 0.2 (variation between log10 for two consecutive measurements is higher than or equal to 0.2). In addition to the analyses above, the dataset of the ONSET and COURSE study provides ample opportunities to look into the relationship between immune parameters, infections, psychosocial stress, genetic factors and tic onsets and exacerbations.

### Genetic analyses

Given the diverse origin of the samples that will be included in the produced data sets, particular attention will be paid to statistical techniques that correct for biases that are typically introduced in such analyses, due to population stratification. To assist in this, we will leverage the power of Principal Components Analysis to detect stratification and population substructure. To uncover gene–environment interactions, the case-only design and traditional (e.g. multi-dimensionality-reduction) as well as machine-learning techniques will be used. The resulting list of target susceptibility variants will be refined through gene-network analysis and search of public databases of gene function and ontology. Quantitative trait loci (QTLs) will be identified among the single nucleotide polymorphisms (SNPs) yielding results of high statistical significance to assist in the identification of possible gene pathways that are involved in the susceptibility to the studied disorders. Public gene expression databases (e.g. HapMap data of gene expression from lymphoblastoid cell lines) will be used to that effect.

We will also mine for gene–gene and gene–environment interactions. In particular, we will attempt to leverage linear discriminant analysis (LDA), a powerful dimensionality reduction technique, to design statistical tests that analyse all the SNP genotypes from a GWAS simultaneously (as opposed to one at the time) and thus identify a subset of SNPs that are most associated or predictive of disease risk. This technique achieves improved performance over single SNP tests.

#### Gene expression analyses

Statistical tests (e.g. *T* tests, ANOVA, ANCOVA) will be performed using Partek, GeneSpring, PAM and BRB-ArrayTools. Special care is going to be taken to correct for potential confounding factors (e.g. ancestry, sex, age). Multiple comparison corrections will be performed using Benjamini and Hochberg False Discovery Rate. A tenfold cross validation will be performed, and the minimum number of genes that predicts a given class will be derived using prediction analysis of microarrays (PAM) which uses a nearest neighbour, smallest shrunken centroid algorithm. Other prediction algorithms including support vector machine methods will be used to confirm these results. Cluster analyses and principal component analyses will be used to visualize how well the genes separate the groups, or to discover new classes.

### Data management and data cleaning

All data were collected on paper and entered into an electronic data base by each site. The data in each site are encrypted and kept in a secured place, and were transmitted in a secure manner, in line with requirements of data protection and data transmission regulations. At each site, a unique code was assigned to recruited members, and tests were delivered to the labs, withholding any identifying information (e.g. name, initials, birth date). After collection of clinical data had been completed we checked all data for improbable values and corrections have been applied where needed by going back to source material. Data access to researchers from outside the EMTICS consortium research shall be granted on fair and reasonable conditions.

### Training

All collaborators were trained in clinical assessments with a focus on the YGTSS by use of video recording of children with tics and in appropriate conduct of research in accordance with ethical requirements. Moreover, throughout the 6.5 years duration of the study we had bi-annual meetings with all investigators in which various aspects of the study were discussed aimed at standardisation of procedures and obtaining agreement. In addition, in case of diagnostic uncertainties, consensus scoring was used within the expert team of the respective clinical centres. The microbiological methods used by different centres to isolate GAS from carriers with low bacterial density were harmonised and improved through a training course and two external quality assessments.

## Discussion

The EMTICS study provides the unique opportunity to analyse data on a broad set of environmental exposures and biological markers related to chronic tic disorders under the umbrella of a common methodological framework. The ONSET cohort of the EMTICS study represents, to the best of our knowledge, the first attempt to capture in a prospective fashion the onset of tics in an at-risk population, i.e. first-degree relatives of probands with a chronic tic disorder. At the same time, the COURSE cohort of the EMTICS study represents, to date, the largest cohort of youth with a chronic tic disorder followed up systematically and in a prospective fashion to capture clinically relevant exacerbations of tics and/or OCD symptoms. It offers therefore the possibility to investigate, with larger power than previously sought, the potential determinants of severity fluctuations within these disorders. This type of design and dataset offers an as yet unforeseen opportunity to develop prediction models and algorithms that quantify the risk of onset or exacerbation of tic disorders based on environmental exposures and gene–environment interactions. Such predictive algorithms would then need further validation in other populations before being introduced in routine clinical activity.

The previous studies that have tackled the multifactorial influence of environment and gene–environment interactions upon the natural history of chronic tic disorders were either population-based studies that used national health- or health insurance claims-based registries, or single- or multicentre, clinic-based, prospective studies. Population-based studies have reported associations of TS/chronic tic disorders and common comorbidities with a very specific environmental exposure such as GAS infections [50–54], with little or no insight on interactions between exposures. Although the majority of these studies reported an increased risk of tic disorders for individuals with a history of previous streptococcal throat infection ranging between 1.2 and 2.2, these estimates vary across studies, and in at least one study [54] the association was not confirmed. The variability across health data registry studies in the definition of GAS infections as well as in the source and methodology of data collection from different care providers possibly reasons for the discrepancies across risk estimates. Our prospective, hypothesis-based study design overcomes this limitation, using a strict and standardized, homogenous and quality-controlled determination of GAS colonisation. Moreover, our study design guarantees homogeneity, compared to population-based studies, also with respect to the assessment of tic disorders and concurrent symptoms through a trained group of clinicians, minimising medical surveillance bias and recall bias. We acknowledge, however, that some of the data collected in EMTICS, e.g. those on pre- and perinatal adversities might also suffer from a certain degree of recall bias, in the lack of supporting medical documentation. Another advantage with respect to previous population-based studies is the possibility for correlating clinically relevant events or exposures to biological measures, which is key to understanding multifactorial interactions and elaborate mechanistic explanations for the observed associations. The main limitation compared to population-based studies is the risk of a larger type II error, particularly in the ONSET cohort, linked to smaller study power.

Previous prospective, clinic-based studies enrolled smaller (*N* < 50 with the exception of [54] patient cohorts [55–59], often aiming to isolate a subgroup of patients with PANDAS from a clinic-based population of patients with a ‘garden variety’ tic disorders. The research questions of the EMTICS study do not refer to the PANDAS or the PANS spectrum, and indeed clinical data gathered in EMTICS are insufficient to diagnose PANS accurately. Compared to previous clinic-based studies, EMTICS relies on rating scale based data to confirm or exclude exacerbations of tics, thus minimising a potential bias resulting from subjective definitions of exacerbation based on the judgement of individual clinicians. Previous clinic-based studies have, in some cases [56, 59], applied a more intensive clinical follow-up over a period of 24 months, although this applied to a population of less than 90 patients. Logistic limitations related to the international composition of the EMTICS list of enrolment sites did not allow such an intensive follow-up for a very large population. We partially mitigated this potential limitation in the COURSE cohort by replacing highly frequent clinic visits with the use of structured weekly diaries and bi-monthly telephone interviews in between clinic visits. A caveat of this logistic limitation is the greater reliance on self-reported data. Finally, prospective clinic-based studies did not analyse in detail interactions between exposures, with the only exception of the study by Lin et al. [59], which evaluated the interaction between GAS infections and psychosocial stress levels on a relatively small population of 45 cases with TS and/or OCD and 41 matched control subjects. The design of EMTICS appears highly promising to test this interaction with a substantially greater statistical power.

Albeit comprehensive, the EMTICS study does not include all the potential pathophysiological biomarkers of TS that could be influenced by environmental exposures and gene–environment interactions, e.g. imaging of brain networks or microbiome data. The EMTICS research platform has, however, included parallel studies of animal models that aim to replicate some of the pathophysiological constructs explored by clinical studies. Some of these animal model data have already been published [83–85] and a detailed explanation of these pre-clinical studies is beyond the scope of this article. We acknowledge that other comorbid behavioural symptoms, e.g. ADHD symptoms, may also fluctuate over time in patients with tic disorders; however, we focused on the prospective evaluation of tics and obsessive–compulsive symptoms, as preliminary studies reported a similar short-term influence of putative environmental risk factors (psychosocial stress, GAS infections) on both tic and obsessive–compulsive symptom severity.

Lastly, one concern regarding the gene expression study in EMTICS has been whether peripheral blood can mirror the neuronal tissue, when it comes to detecting altered gene expression profiles that could be relevant to brain pathology. However, recent data support the fact that changes of gene expression in blood may actually constitute distinct molecular signatures of not only clearly hereditary disorders (e.g. neurofibromatosis type I, tuberous sclerosis complex 2, Down’s syndrome [86]; Huntington’s disease Borovecki [87]), but also complex disorders of the brain (multiple sclerosis [88]; migraine and TS [89, 90]). Thus, there has been considerable effort in determining whether blood gene expression profiling can provide surrogate markers for neurological diseases, including TS, especially when considering (1) the inaccessibility of the actual diseased tissue, and (2) the often very young age of cases with TS participating in research studies, such as EMTICS. In fact, it was recently shown that distinct gene expression changes were correlated with tic severity in medicated versus unmedicated TS cases [91], whereas specific molecular signatures were correlated with inattention and hyperactivity/impulsivity, a very common TS comorbidity, in individuals with TS [92]. Therefore, we believe that EMTICS may further elucidate the genetic background underlying tic disorders and to may shed light on how these interact with environmental factors influencing the onset and clinical course of tic disorders.

We conclude that this study, the first European study of TS of this size granted by the EU, provides innovative and unique avenues to address the aetiology of chronic tic disorders, and we hope that it will represent the consolidation of a stable collaborative research and clinical environment on TS and related disorders in Europe.

### Electronic supplementary material

Below is the link to the electronic supplementary material.

Supplementary material 1 (PDF 641 kb)

## Appendix

The EMTICS group members are Zacharias Anastasiou^1^, Alan Apter^2^, Valentina Baglioni^3^, Juliane Ball^4^, Erika Bartolini^5^, Noa Benaroya-Milshtein^2^, Benjamin Bodmer^6^, Emese Bognar^7^, Bianka Burger^8,9^, Judith Buse^6^, Maura Buttiglione^10^, Francesco Cardona^3^, Marta Correa Vela^11^, Roberta Creti^12^, Andrea Dietrich^13^, Nanette M. Debes^14^, Androulla Efstratiou^15^, Maria Cristina Ferro^16^, Carolin Fremer^17^, Blanca Garcia-Delgar^18^, Maria Gariup^19,20^, Marianthi Georgitsi^21,22^, Mariangela Gulisano^16^, Annelieke Hagen^23,24^, Julie Hagstrøm^25^, Tammy J. Hedderly^26^, Isobel Heyman^27^, Pieter J. Hoekstra^13^, Chaim Huyser^23,24^, Monica Imperi^12^, Iordanis Karagiannidis^21^, Giovanni Laviola^28^, Simone Macri^28^, Marcos Madruga-Garrido^29^, Immaculada Margarit^5^, Anna Marotta^30^, Davide Martino^31^, Ute C. Meier^32^, Pablo Mir^11^, Natalie Moll^33^, Astrid Morer^18,34,35^, Norbert Müller^8,9^, Kirsten Müller-Vahl^17^, Alexander Münchau^36^, Peter Nagy^7^, Valeria Neri^3^, Thaïra J.C. Openneer^13^, Graziella Orefici^37^, Peristera Paschou^38^, Alessandra Pellico^16^, Onofrio Petruzzelli^10^, Kerstin J. Plessen^25,39^, Cesare Porcelli^30^, Marina Redondo^18^, Renata Rizzo^16^, Paolo Roazzi^40^, Veit Roessner^6^, Daphna Ruhrman^2^, Jaana M.L. Schnell^8^, Anette Schrag^41^, Gregor A. Schütze^33^, Markus J. Schwarz^33^, Paola Rosaria Silvestri^3^, Liselotte Skov^42^, Tamar Steinberg^2^, Sara Stöber^42^, Friederike Tagwerker Gloor^4^, Marco Tallon^43^, Zsanett Tarnok^7^, Victoria L. Turner^26^, Susanne Walitza^4^, Elif Weidinger^8^, and Martin L. Woods^26^

^1^Department of Statistical Science, University College London, London, UK

^2^Child and Adolescent Psychiatry Department, Schneider Children’s Medical Center of Israel, affiliated to Sackler Faculty of Medicine, Tel Aviv University, Petah-Tikva, Israel

^3^University La Sapienza of Rome, Department of Human Neurosciences, Rome, Italy

^4^Clinic of Child and Adolescent Psychiatry and Psychotherapy, University of Zurich, Zurich, Switzerland

^5^GSK, Siena, Italy

^6^Department of Child and Adolescent Psychiatry, Faculty of Medicine of the TU Dresden, Dresden, Germany

^7^Vadaskert Child and Adolescent Psychiatric Hospital, Budapest, Hungary

^8^Department of Psychiatry and Psychotherapy, University Hospital, LMU Munich, Munich, Germany

^9^Marion von Tessin Memory-Zentrum gGmbH, Munich, Germany

^10^University of Bari “Aldo Moro”, Medical School, Department of Biological Sciences and Human Oncology, Bari, Italy

^11^Unidad de Trastornos del Movimiento, Servicio de Neurología y Neurofisiología Clinica. Instituto de Biomedicina de Sevilla (IBiS), Hospital Universitario Virgen del Rocio/CSIC/Universidad de Sevilla, Seville, Spain

^12^Department of Infectious Diseases, Istituto Superiore di Sanità, Rome, Italy

^13^University of Groningen, University Medical Center Groningen, Department of Child and Adolescent Psychiatry, Groningen, the Netherlands

^14^Paediatric Department, Herlev University Hospital, Herlev, Denmark

^15^WHO Global Collaborating Centre for Reference and Research on Diphtheria and Streptococcal Infections; Reference Microbiology, Directorate National Infection Service, Public Health England, London, UK

^16^Child Neuropsychiatry Section, Department of Clinical and Experimental Medicine, School of Medicine, Catania University, Catania, Italy

^17^Clinic of Psychiatry, Socialpsychiatry and Psychotherapy, Hannover Medical School, Hannover, Germany

^18^Department of Child and Adolescent Psychiatry and Psychology, Institute of Neurosciences, Hospital Clinic Universitari, Barcelona, Spain

^19^University of Barcelona, Barcelona, Spain

^20^Copenhagen Psychiatric Center, Intensive Inpatient Unit, Copenhagen, Denmark

^21^Department of Molecular Biology and Genetics, Democritus University of Thrace, Alexandroupoli, Greece

^22^Department of Medicine, Aristotle University of Thessaloniki, Thessaloniki, Greece

^23^De Bascule, Academic Center for Child and Adolescent Psychiatry, Amsterdam, the Netherlands

^24^Academic Medical Center, Department of Child and Adolescent Psychiatry, Amsterdam, the Netherlands

^25^Child and Adolescent Mental Health Center, Mental Health Services, Capital Region of Denmark and University of Copenhagen, Copenhagen, Denmark

^26^Evelina London Children’s Hospital GSTT, Kings Health Partners AHSC, London, UK

^27^Great Ormond Street Hospital for Children, and UCL Institute of Child Health, London, UK

^28^Reference Centre for Behavioural Sciences and Mental Health, Istituto Superiore di Sanità, Roma, Italy

^29^Sección de Neuropediatría, Instituto de Biomedicina de Sevilla (IBiS), Hospital Universitario Virgen del Rocío/CSIC/Universidad de Sevilla, Seville, Spain

^30^Azienda Sanitaria Locale di Bari, Mental Health Department, Child and Adolescent Service of Bari Metropolitan Area, Bari, Italy

^31^Department of Clinical Neurosciences, University of Calgary, Calgary, Canada

^32^Blizard Institute, Queen Mary University of London, London, UK

^33^Institute of Laboratory Medicine, University Hospital, LMU Munich, Munich, Germany

^34^Institut d’Investigacions Biomediques August Pi i Sunyer (IDIBAPS), Barcelona, Spain

^35^Centro de Investigacion en Red de Salud Mental (CIBERSAM), Instituto Carlos III, Spain

^36^Institute of Neurogenetics, University of Lübeck, Lübeck, Germany

^37^formerly Department of Infectious Diseases, Istituto Superiore di Sanità, Rome, Italy

^38^Department of Biological Sciences, Purdue University, West Lafayette, USA

^39^Service of Child and Adolescent Psychiatry, Department of Psychiatry, University Medical Center, University of Lausanne, Lausanne, Switzerland

^40^Health Technology Assessment Centre, Istituto Superiore di Sanità, Rome, Italy

^41^Department of Clinical Neuroscience, UCL Institute of Neurology, University College London, London, UK

^42^Concentris research management GmbH, Fürstenfeldbruck, Germany

^43^IT Service, Istituto Superiore di Sanità, Rome, Italy

Laboratory centres and research management:

^1^Advanced Practical Diagnostics (ApDia), Belgium (Raf Berghmans)

^2^deCODE Genetics, Reykjavik, Iceland

^3^Biolytics, Witterswil, Switzerland

^4^concentris research management GmbH, Germany (Ameli Schwalber, Sara Stöber)

^5^Cytolab, Switzerland (Adrian Urwyler)

^6^GSK Vaccines, Siena, Italy (Immaculada Margarit, Erika Bartolini)

^7^Health Technology Assessment Centre, National Institute of Health (Istituto Superiore di Sanità, ISS), Italy (Paolo Roazzi)

^8^LMU Munich, Germany (Markus Schwarz)

^9^University of Groningen, University Medical Center Groningen, Netherlands (coordinating site, Pieter J. Hoekstra)

^10^ProImmune, UK (Andrew Waller, Nikolai Schwabe)

^11^TU Dresden, Germany (Veit Roessner)

^12^University of Bari “Aldo Moro”, Italy (Maura Buttiglione)
