# Supplementary material for: European Multicentre Tics in Children Studies (EMTICS): protocol for two cohort studies to assess risk factors for tic onset and exacerbation in children and adolescents
Source: Eur Child Adolesc Psychiatry. 2018 Jul 7;28(1):91–109. doi: 10.1007/s00787-018-1190-4 (PMC6349795; doi:10.1007/s00787-018-1190-4)
Supplement: Supplementary file 1 — Supplementary material 1 (PDF 641 kb) [file 787_2018_1190_MOESM1_ESM.pdf]

SUPPLEMENTARY MATERIALS

Supplementary Table 1. Assessment schedule for the ONSET study of the European Multicentre Tics in Children Studies (EMTICS) with interchanging 4-monthly telephone interviews and hospital visits.

**Week 0**	**Baseline**
Weeks 1-7	Weekly diaries 1-7
**Week 8**	**Tele 1**
Weeks 9-15	Weekly diaries 8-14
**Week 16**	**Follow-up visit 1**
Weeks 17-23	Weekly diaries 15-21
**Week 24**	**Tele 2**
Weeks 25-31	Weekly diaries 22-28
**Week 32**	**Follow-up visit 2**
Weeks 33-39	Weekly diaries 29-35
**Week 40**	**Tele 3**
Weeks 41-47	Weekly diaries 36-42
**Week 48**	**Follow-up visit 3**
Weeks 49-55	Weekly diaries 43-49
**Week 56**	**Tele 4**
Weeks 57-63	Weekly diaries 50-56
**Week 64**	**Follow-up visit 4**
Weeks 65-71	Weekly diaries 57-63
**Week 72**	**Tele 5**
Weeks 73-79	Weekly diaries 64-70

Follow up Supplement Table 1

**Week 80**	**Follow-up visit 5**
Weeks 81-87	Weekly diaries 71-77
**Week 88**	**Tele 6**
Weeks 89-95	Weekly diaries 78-84
**Week 96**	**Follow-up visit 6**
Weeks 97-103	Weekly diaries 85-91
**Week 104**	**Tele 7**
Weeks 105-111	Weekly diaries 92-98
**Week 112**	**Follow-up visit 7**
Weeks 113-119	Weekly diaries 99-105
**Week 120**	**Tele 8**
Weeks 121-127	Weekly diaries 106-112
**Week 128**	**Follow-up visit 8**
Weeks 129-135	Weekly diaries 113-119
**Week 136**	**Tele 9**
Weeks 137-143	Weekly diaries 120-126
**Week 144**	**Final visit**

Tele, telephone interview. This schedule represents the situation when there is no tic onset during the 3-year study period.

Supplementary Table 2. Assessment schedule for the COURSE study of the European Multicentre Tics in Children Studies (EMTICS) with interchanging 4-monthly telephone interviews and hospital visits.

**Week 0**	**Baseline**
Weeks 1-7	Weekly diaries 1-7
**Week 8**	**Tele 1**
Weeks 9-15	Weekly diaries 8-14
**Week 16**	**Follow-up visit 1**
Weeks 17-23	Weekly diaries 15-21
**Week 24**	**Tele 2**
Weeks 25-31	Weekly diaries 22-28
**Week 32**	**Follow-up visit 2**
Weeks 33-39	Weekly diaries 29-35
**Week 40**	**Tele 3**
Weeks 41-47	Weekly diaries 36-42
**Week 48**	**Follow-up visit 3**
Weeks 49-55	Weekly diaries 43-49
**Week 56**	**Tele 4**
Weeks 57-63	Weekly diaries 50-56
**Week 64**	**Final visit**

Tele, telephone interview. This schedule represents the situation when there is no tic exacerbation during the 16-months study period.

Supplementary Table 3. Overview of all different possible tic exacerbation scenarios of the COURSE study. The protocol allowed for the identification of a maximum number of two pairs of expedited exacerbation and post-exacerbation visits per participating child. Per protocol, no additional expedited visit could be scheduled before completion of the post exacerbation expedited visit, which was scheduled two months after an expedited tic exacerbation visit. Thus, each child will have a maximum of five clinic visits, regardless if visits were expedited or not. The only exceptions are scenarios #5 and #7 with six visits and a maximum study duration of 18 months rather than 16 months. Note that as tic exacerbation visits are expedited and may occur earlier than the next planned visit or telephone interview, study duration was shortened accordingly. Telephone interviews were always scheduled in the midst of the 4 months' intervals (not shown here).

Scenario 1: *no tic exacerbations at all*

Baseline visit	
Follow-up visit 1	4 months after baseline visit
Follow-up visit 2	4 months after follow-up visit 1
Follow-up visit 3	4 months after follow-up visit 2
Final visit	4 months after follow-up visit 3

Scenario 2: *one tic exacerbation (identified before or at planned follow-up visit 1)*

Baseline visit	
Expedited visit (tic exacerbation)	≤ 4 months after baseline visit
Post-exacerbation expedited visit	2 months after expedited visit
Follow-up visit 3	4 months after post-exacerbation expedited visit
Final visit	4 months after follow-up visit 3

Scenario 3: *one tic exacerbation (between follow-up visit 1 and before or at planned follow-up visit 2)*

Baseline visit	
Follow-up visit 1	4 months after baseline visit
Expedited visit (tic exacerbation)	≤ 4 months after follow-up visit 1
Post-exacerbation expedited visit	2 months after expedited visit
Final visit	4 months after post-exacerbation expedited visit

Scenario 4: *one tic exacerbation (between follow-up visit 2 and before or at planned follow-up visit 3)*

Baseline visit	
Follow-up visit 1	4 months after baseline visit
Follow-up visit 2	4 months after follow-up visit 1
Expedited visit (tic exacerbation)	≤ 4 months after follow-up visit 2
Combined final and post-exacerbation expedited visit*	2 months after expedited visit

Scenario 5: *one tic exacerbation (between follow-up visit 3 and before or at planned final visit)*

Baseline visit	
Follow-up visit 1	4 months after baseline visit
Follow-up visit 2	4 months after follow-up visit 1
Follow-up visit 3	4 months after follow-up visit 2
Expedited visit (tic exacerbation)	≤ 4 months after follow-up visit 3
Combined final and post-exacerbation expedited visit*	2 months after expedited visit

Scenario 6: *two tic exacerbations*

Baseline visit	
Expedited visit (tic exacerbation) 1	≤ 4 months after baseline visit
Post-exacerbation expedited visit 1	2 months after expedited visit 1
Expedited visit (tic exacerbation) 2	≤ 4 months after post-exacerbation expedited visit 1
Combined final and post-exacerbation expedited visit*	2 months after expedited visit 2

Scenario 7: *two tic exacerbations*

Baseline visit	
Follow-up visit 1	4 months after baseline visit
Expedited visit (tic exacerbation) 1	≤ 4 months after follow-up visit 1
Post-exacerbation expedited visit 1	2 months after expedited visit 1
Expedited visit (tic exacerbation) 2	≤ 4 months after post-exacerbation expedited visit 1
Combined final and post-exacerbation visit*	2 months after expedited visit 2

* Measurements of the final visit and the post-exacerbation expedited visit were combined.

Title: European Multicentre Tics in Children Studies (EMTICS): Protocol for two cohort studies to assess risk factors and aetiology of tics in children and adolescents; Journal: European Child and Adolescent Psychiatry; *Authors: Schrag A, Martino D et al; Corresponding author: Hoekstra PJ, University of Groningen, University Medical Center Groningen, Department of Child and Adolescent Psychiatry, Hanzeplein 1, 9713 GZ Groningen, The Netherlands, p.hoekstra@accare.nl.*
